# Comparative Genome Analyses of *Streptococcus suis* Isolates from Endocarditis Demonstrate Persistence of Dual Phenotypic Clones

**DOI:** 10.1371/journal.pone.0159558

**Published:** 2016-07-19

**Authors:** Mari Tohya, Takayasu Watanabe, Fumito Maruyama, Sakura Arai, Atsushi Ota, Taryn B. T. Athey, Nahuel Fittipaldi, Ichiro Nakagawa, Tsutomu Sekizaki

**Affiliations:** 1 Research Center for Food Safety, Graduate School of Agricultural and Life Sciences, The University of Tokyo, Bunkyo-ku, Tokyo, Japan; 2 Department of Microbiology, Kyoto University Graduate School of Medicine, Kyoto, Kyoto, Japan; 3 Public Health Ontario, Toronto, Ontario, Canada; 4 Department of Laboratory Medicine and Pathobiology, Faculty of Medicine, University of Toronto, Toronto, Ontario, Canada; University of Iowa Carver College of Medicine, UNITED STATES

## Abstract

Many bacterial species coexist in the same niche as heterogeneous clones with different phenotypes; however, understanding of infectious diseases by polyphenotypic bacteria is still limited. In the present study, encapsulation in isolates of the porcine pathogen *Streptococcus suis* from persistent endocarditis lesions was examined. Coexistence of both encapsulated and unencapsulated *S*. *suis* isolates was found in 26 out of 59 endocarditis samples. The isolates were serotype 2, and belonged to two different sequence types (STs), ST1 and ST28. The genomes of each of the 26 pairs of encapsulated and unencapsulated isolates from the 26 samples were sequenced. The data showed that each pair of isolates had one or more unique nonsynonymous mutations in the *cps* gene, and the encapsulated and unencapsulated isolates from the same samples were closest to each other. Pairwise comparisons of the sequences of *cps* genes in 7 pairs of encapsulated and unencapsulated isolates identified insertion/deletions (indels) ranging from one to 10^4^ bp in different *cps* genes of unencapsulated isolates. Capsule expression was restored in a subset of unencapsulated isolates by complementation *in trans* with *cps* expression vectors. Examination of gene content common to isolates indicated that mutation frequency was higher in ST28 pairs than in ST1 pairs. Genes within mobile genetic elements were mutation hot spots among ST28 isolates. Taken all together, our results demonstrate the coexistence of dual phenotype (encapsulated and unencapsulated) bacterial clones and suggest that the dual phenotypes arose independently in each farm by means of spontaneous mutations in *cps* genes.

## Introduction

Bacteria can adapt to environmental changes through various adaptive strategies. One example is coexistence of different phenotypes of the same organism at specific niches [1]. Polyphenotypic infections by a single species have been reported in many bacterial species, including *Pseudomonas aeruginosa*, *Helicobacter pylori*, *Escherichia coli*, and *Staphylococcus epidermidis* [1–4]. The ecologies of these infections have shown that the composition of polyphenotypic clones permit adaptation to various environmental conditions. Coexistence by polyphenotypic clones is thus thought to be a barrier to treatment and prophylaxis [2, 4–7]. On the other hand, the compositions of coexistable bacteria are affected by antimicrobials and host immunity [8, 9]. Therefore, bacterial population dynamics in response to environmental changes play an important role in evasion from host immune system [10–12].

Variation of surface structures in bacteria is important for adaptation and survival in variable environments. Although capsules are major bacterial extracellular components and show antiphagocytic effect, the loss of capsules can also be beneficial [13–16]. Hanage *et al*. reported that one or more capsule-related genes were not detected by PCR in isolates of *Streptococcus pneumoniae* from nasopharyngeal swabs and middle ear fluid [10]. This finding suggested that unencapsulation afforded advantages to capsule-less clones in some habitats. In *Streptococcus pyogenes*, unencapsulated clones isolated from pharyngitis and invasive diseases are known to be invasive and cause diseases [16, 17]. Similarly, unencapsulated clones of the zoonotic pathogen *Streptococcus suis* [18–20] were isolated from porcine endocarditis lesions [21]. The role of these phenotypic changes has, however, not yet been established. In addition, although *S*. *suis* isolates from different worldwide locations are known to be genetically highly diverse, there is no information about the genetic diversity of isolates recovered from a single lesion [22].

The *S*. *suis* capsule is composed of capsular polysaccharides (CPs), whose biosynthesis is dependent on the concerted action of enzymes encoded by genes located in the so-called capsular polysaccharide synthesis (*cps*) locus [23–27]. Different antigenicities of the *S*. *suis* CPs are the basis of *S*. *suis* serotyping [27, 28]. *S*. *suis* CPs have been shown to play a major role in protection against host phagocytes [29–31], and unencapsulated *S*. *suis* clones have been described as low virulent or completely avirulent, leading to the notion that loss of capsule is unfavorable for *S*. *suis* virulence. However, the ability to form biofilms and to adhere to epithelial cells and platelets was previously reported to be stronger in unencapsulated clones than in encapsulated ones [32–34], as unencapsulated clones exclusively invaded into epithelial cells [32, 34, 35]. Therefore, from an ecological perspective, it is plausible that unencapsulated and encapsulated *S*. *suis* clones coexist in the same lesions, and that the presence of both encapsulated and unencapsulated cells originated from genetically identical clones facilitate adherence and invasion to host cells, thereby permitting efficient evasion from the immune system and persistence of the clonal population in a particular habitat or niche.

In the present study, to determine whether encapsulated and unencapsulated, or dual phenotypic *S*. *suis* clones coexist in porcine endocarditis lesions, we collected multiple *S*. *suis* isolates from each endocarditis lesion and confirm encapsulation status of the isolates. Finally, comparative genome analyses allowed us to precisely define the close relationships between encapsulated and unencapsulated isolates from the same endocarditis lesion. Our findings suggest that mutations from encapsulated cells to unencapsulated ones occurred independently in each pig or farm.

## Materials and Methods

### Bacteria, plasmids and growth condition

**Fig 1:**
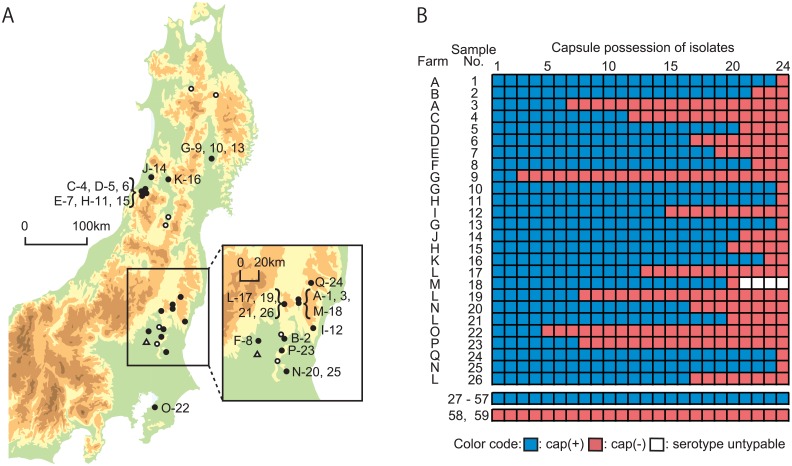
Geographic location of porcine farms examined and results of encapsulated and unencapsulated *S*. *suis* isolates. (A) The map shows the northern east area of the main island of Japan from which *S*. *suis* was isolated in the present study. The farms are indicated by the following symbols: blank circles and blank triangles: encapsulated and unencapsulated *S*. *suis*, respectively, were exclusively isolated, and filled circles: encapsulated and unencapsulated *S*. *suis* were both isolated. The farm identifiers (alphabetical characters) and sample Nos. of endocarditis samples are indicated along the above symbols. The map was publicly available from the Geospatial Information Authority in Japan. (B) The heat map shows the encapsulation traits of 24 *S*. *suis* isolates in each of 59 endocarditis samples, with the following colors: blue for encapsulated, and red for unencapsulated isolates. Untypeable isolates in serotype-specific PCR are indicated by blanks. Encapsulated and unencapsulated isolates were exclusively from 31 (Nos. 27–57) and 2 (Nos. 58 and 59) endocarditis samples, respectively. Sample Nos. 1 to 26 were hereafter used as the No. of the pair of encapsulated and unencapsulated isolates.

Fifty-nine heart valve vegetations of porcine endocarditis from 24 farms (Fig 1A) were collected in meat inspection centers in Japan between 2013 and 2014. These samples were stamped onto Todd-Hewitt (TH, Becton Dickinson, MD, U.S.) agar plates containing 5% horse blood, followed by incubation at 37°C for 16 hours under a 5% CO_2_ atmosphere. Twenty-four colonies were picked from each sample and purified twice by single colony isolation. Amplification of the *S*. *suis recN* gene, which serves to confirm species [36] was performed by PCR, and the positive isolates were further examined by PCR permitting serotype estimation [37, 38]. All isolates were stored in Luria-Bertani (LB, Becton Dickinson) broth containing 30% glycerol at -80°C. For *S*. *suis* isolates, encapsulation was determined using a co-agglutination test [21]. One pair of encapsulated and unencapsulated *S*. *suis* isolates was selected from each of 26 endocarditis samples that yielded both encapsulation and unencapsulated phenotypes (a total of 52 isolates, Table 1). *Escherichia coli* strains and plasmids used in this study are listed in S1 Table [39, 40]. *E*. *coli* strains were grown on LB broth at 37°C for 16 hours. When necessary, media were supplemented with spectinomycin (50 μg/ml or 100 μg/ml for *E*. *coli* and *S*. *suis*, respectively).

**Table 1:** *S*. *suis* isolates from Japan used in this study.

Pair No.	Isolate No.		Farm	Location	Serotype	MLST	Accession No. of DRA*	
	Encapsulated	Unencapsulated					Encapsulated	Unencapsulated
1	SUT709	SUT708	A	Nasu-gun, Tochigi	2	ST28	DRX045114	DRX045113
2	SUT780	SUT785	B	Utsunomiya-shi, Tochigi	2	ST28	DRX045115	DRX045116
3	SUT806	SUT804	A	Nasu-gun, Tochigi	2	ST28	DRX045118	DRX045117
4	SUT877	SUT876	C	Tsuruoka-shi, Yamagata	2	ST1	DRX045120	DRX045119
5	SUT906	SUT907	D	Tsuruoka-shi, Yamagata	2	ST1	DRX045121	DRX045122
6	SUT926	SUT927	D	Tsuruoka-shi, Yamagata	2	ST1	DRX045123	DRX045124
7	SUT1006	SUT1007	E	Tsuruoka-shi, Yamagata	2	ST28	DRX045125	DRX045126
8	SUT1020	SUT1024	F	Shimotsuga-gun, Tochigi	2	ST1	DRX045127	DRX045128
9	SUT1046	SUT1044	G	Tanzawa-gun, Iwate	2	ST28	DRX045130	DRX045129
10	SUT1189	SUT1190	G	Tanzawa-gun, Iwate	2	ST28	DRX045131	DRX045132
11	SUT1330	SUT1329	H	Tsuruoka-shi, Yamagata	2	ST28	DRX045134	DRX045133
12	SUT1337	SUT1334	I	Hitachiomiya-shi, Ibaraki	2	ST28	DRX045136	DRX045135
13	SUT1468	SUT1467	G	Tanzawa-gun, Iwate	2	ST28	DRX045138	DRX045137
14	SUT1582	SUT1583	J	Shonai-shi, Yamagata	2	ST28	DRX045139	DRX045140
15	SUT1596	SUT1607	H	Tsuruoka-shi, Yamagata	2	ST28	DRX045141	DRX045142
16	SUT1678	SUT1675	K	Mogami-gun, Yamagata	2	ST28	DRX045144	DRX045143
17	SUT1789	SUT1797	L	Nasukarasuyama-shi, Tochigi	2	ST28	DRX045145	DRX045146
18	SUT1865	SUT1882	M	Nasu-machi, Nasu-gun, Tochigi	2	ST28	DRX045147	DRX045148
19	SUT1914	SUT1908	L	Nasukarasuyama-shi, Tochigi	2	ST1	DRX045150	DRX045149
20	SUT2052	SUT2055	N	Ishioka-shi, Ibaraki	2	ST28	DRX045151	DRX045152
21	SUT2083	SUT2080	L	Nasukarasuyama-shi, Tochigi	2	ST28	DRX045154	DRX045153
22	SUT2148	SUT2154	O	Ichihara-shi, Chiba	2	ST28	DRX045155	DRX045156
23	SUT2188	SUT2189	P	Moka-shi, Tochigi	2	ST28	DRX045157	DRX045158
24	SUT2199	SUT2210	Q	Higashishirakawa-gun, Fukushima	2	ST1	DRX045159	DRX045160
25	SUT2222	SUT2241	N	Ishioka-shi, Ibaraki	2	ST28	DRX045161	DRX045162
26	SUT2256	SUT2250	L	Nasukarasuyama-shi, Tochigi	2	ST28	DRX045164	DRX045163

* DRA, the DNA Data Bank of Japan (DDBJ) Sequence Read Archive

### Genomic DNA extraction

Genomic DNA was extracted from the 52 *S*. *suis* isolates as described previously [41]. DNA concentrations were determined using the Quant-iT PicoGreen dsDNA Assay Kit (Life Technologies Corporation, CA, U.S.), and quality was checked using a NanoDrop 1000 instrument (Thermo Fisher Scientific, DE, U.S.).

### Multilocus sequence typing (MLST)

The 52 *S*. *suis* isolates were examined by MLST, as described previously [42]. The sequence type (ST) was determined by comparing the nucleotide sequences of 7 loci with the data deposited in the *S*. *suis* MLST database (http://ssuis.mlst.net).

### Whole-genome sequencing

Genomic libraries were prepared using Nextera XT DNA kits (Illumina Inc., CA, U.S.). Paired-end sequencing was performed using MiSeq Reagent Kit v3 (600-cycles) in the Illumina MiSeq platform. Quality trimming and filtering of the obtained sequence reads were performed using CLC Genomics Workbench v8.0.1 (CLC bio, Aarhus, Denmark) with the following parameters: Quality Limit = 0.01, Adapters Trimming = Yes, Remove 5′ Terminal Nucleotides = Yes, Number of 5′ Terminal Nucleotides to Remove = 20, Remove 3′ Terminal Nucleotides = Yes, Number of 3′ Terminal Nucleotides to Remove = 5, and Discard Reads below Length = 50.

### Construction of phylogenetic trees

The preprocessed reads were *de novo* assembled using A5-miseq, as of May 22th, 2015, with default parameters [43]. A maximum parsimony phylogenetic tree for the 52 *S*. *suis* isolates was constructed using kSNP3 v3.0 with a *k*-mer length of 19 [44]. kSNP3 was also used to construct maximum parsimony phylogenetic trees for 12 ST1 isolates with *k*-mer 19 and for 40 ST28 isolates with *k*-mer 21. The Kchooser program in kSNP3 was used to estimate these optimum *k*-mer values. Trees were visualized using FigTree v1.4.2 (http://tree.bio.ed.ac.uk/software/figtree).

### Sequence determination of *cps* genes by Sanger sequencing

The nucleotide sequences of the *cps* genes of 7 pairs of encapsulated and unencapsulated isolates (pair no. 1, 2, 3, 9, 10, 11 and 15) were determined as described previously [21]. Sequencher v4.8 and Molecular Evolutionary Genetics Analysis (MEGA) v5 [45] were used for sequence alignment and detection of mutations, respectively.

### Complementation analysis

In order to construct *cps2E* and *cps2H* expression vectors, we individually amplified the *cps2E* and *cps2H* genes by PCR using genomic DNA of encapsulated *S*. *suis* isolates as template, primers listed in S2 Table, and PrimeSTAR Max DNA Polymerase (TaKaRa Bio, Shiga, Japan). Expression plasmid pMX1 [40] was digested with EcoRΙ (TaKaRa Bio), and then fused with either *cps2E* or *cps2H* fragments using the In-Fusion HD Cloning Kit (TaKaRa Bio). Insert orientation and nucleotide sequences of cloned genes were verified by PCR and Sanger sequencing. The resultant expression vectors, pCps2E and pCps2H, were introduced into unencapsulated *S*. *suis* isolates by electroporation. Restoration of capsule expression was examined by the co-agglutination test as described previously [21, 46].

### Detection of nucleotide mutations

Contigs assembled by A5-miseq for the 52 isolates were annotated using Prokka v1.11, run with default parameters [47]. The pan-genomes analysis pipeline (PGAP) v1.02 [48], run with default parameters, was used for the identification of single-copy common genes that were present in the genomes of all isolates. Single-copy common genes were identified independently for ST1 and ST28. For identification of mobile genetic elements (MGEs), the positions of MGEs in the genome of reference strain NSUI002 [49] were used. PHAST [50] and IslandViewer 3 [51], run with default parameters, were also used to assess MGE contents. The preprocessed (as described above) short-read genome data of 12 ST1 and 40 ST28 isolates were mapped against the P1/7 (ST1) and NSUI002 (ST28) complete genome sequences, respectively, using CLC Genomics Workbench. Mapping was performed with the following parameters: Mismatch Cost = 2, Affine Gap Cost = Yes, Insertion Open Cost = 6, Insertion Extend Cost = 1, Deletion Open Cost = 6, Deletion Extend Cost = 1, Length Fraction = 0.5, Similarity Fraction = 0.8, Auto-Detect Paired Distance = Yes, and Non-Specific Match Handling = Map Randomly. Mutation calling was performed using the Basic Variant Detection tool in the CLC Genomics Workbench with the following parameters: Ignore Broken Pairs = Yes, Minimum Coverage = 10, Minimum Count = 10, Minimum Frequency (%) = 80, Base Quality Filter = Yes, Neighborhood Radius = 5, Minimum Central Quality = 30, Minimum Neighborhood Quality = 25, Relative Read Direction Filter = Yes, and Significance = 1.0% [52, 53]. In each pair of encapsulated and unencapsulated isolates, mutations with nonsynonymous amino acid substitutions in the *cps* genes and other single-copy common genes were identified and were visualized by bar graphs with genome structure of the ST28 reference strain using R v3.2.2. The *cps* gene cluster was included in this comparison even though *cp*s genes had been excluded from pool of common genes used in precious analysis due to presence of the indels spanning over vast nucleotide stretches. The mutation frequency for each single-copy common gene in each ST was calculated as the rate of the number of nucleotides with a mutation divided by the total number.

### Data access

The genome sequence data obtained in this study were deposited in the DNA Data Bank of Japan (DDBJ) Sequence Read Archive under accession number DRA004206.

## Results

### Coexistence of encapsulated and unencapsulated *S*. *suis* in 26 endocarditis samples

A total of 1,416 isolates from 59 endocarditis samples were identified to be *S*. *suis*. In 58 of the endocarditis samples, PCR genotyping showed that 1,388 isolates were serotype 2, and 4 isolates were untypeable, whereas the 24 isolates from the remaining endocarditis sample were serotype 16. On the basis of the co-agglutination test, 31 (53%) and 2 (3%) of the 59 samples, contained encapsulated only, or unencapsulated only isolates, respectively. On the other hand, both encapsulated and unencapsulated isolates were found in 26 samples (44%). Encapsulated and unencapsulated phenotypes were observed in 17 out of the 24 farms examined (70.8%) (Fig 1A). The ratio of encapsulated to unencapsulated isolates varied (Fig 1B).

### Phylogenetic relationships of encapsulated and unencapsulated *S*. *suis* isolates

**Fig 2:**
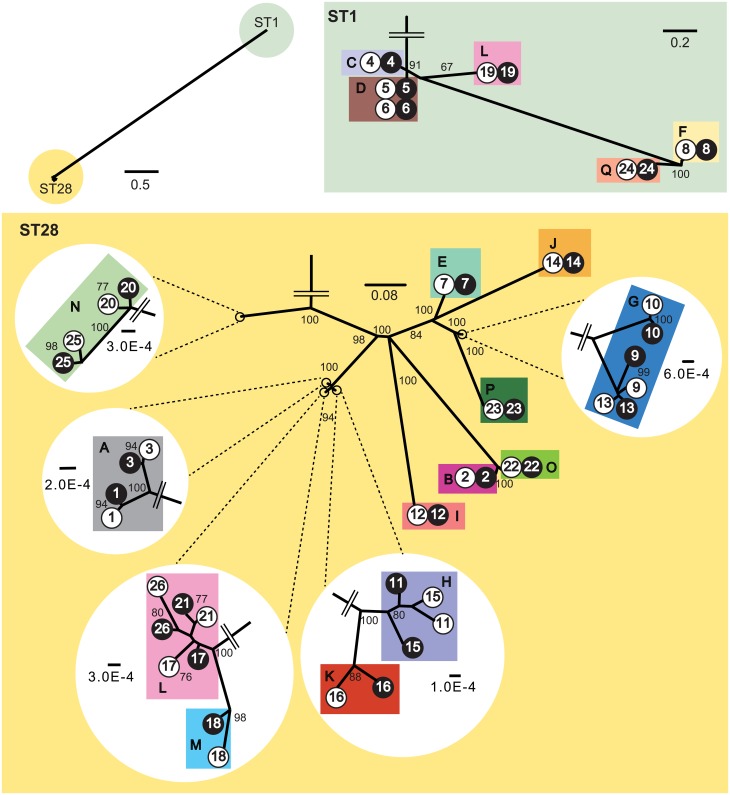
A phylogenetic tree for 26 encapsulated and 26 unencapsulated *S*. *suis* isolates. The tree was constructed using the maximum parsimony method for genome-wide SNPs in 52 *S*. *suis* isolates. The 52 isolates analyzed were composed of 26 encapsulated and unencapsulated isolate pairs from 17 porcine farms, indicated by the following symbols: blank circles for encapsulated, and filled circles for unencapsulated isolates, with pair Nos. and farm identifiers (colored rectangles) along with the symbols. The isolates were separately clustered into two STs, ST1 and ST28; a detailed tree structure in each ST is shown as an enlarged tree. Several ends of the enlarged tree in ST28 are further enlarged. Seventeen colors are used to show distribution of farms in the tree. Only the bootstrap values >50% are indicated on the branches.

We selected a pair of encapsulated and unencapsulated *S*. *suis* isolates from each of the 26 endocarditis samples in order to examine their phylogenetic relationships. The STs of encapsulated and unencapsulated isolates were identical in each of 20 pairs of ST28, and 6 pairs of ST1. Whole-genome short-read data was assembled using A5-miseq (S3 Table presents statistics of the assemblies). Maximum parsimony trees were constructed using genome-wide single-nucleotide polymorphisms (SNPs) compared with the reference stains. In the tree of all 52 isolates, ST1 and ST28 isolates were separately clustered (Fig 2). In each pair of ST1 isolates, the encapsulated and unencapsulated isolates belonged to the same lineage and were phylogenetically distinct from other pairs, except for pairs 5 and 6, which were isolated from the same farm D (Fig 2). In each pair of ST28 isolates, the two were phylogenetically closely related to each other, except for pairs 11 and 15 (Fig 2).

### Mutations in a *cps* gene cluster and complementation with *cps* expression vectors

**Fig 3:**
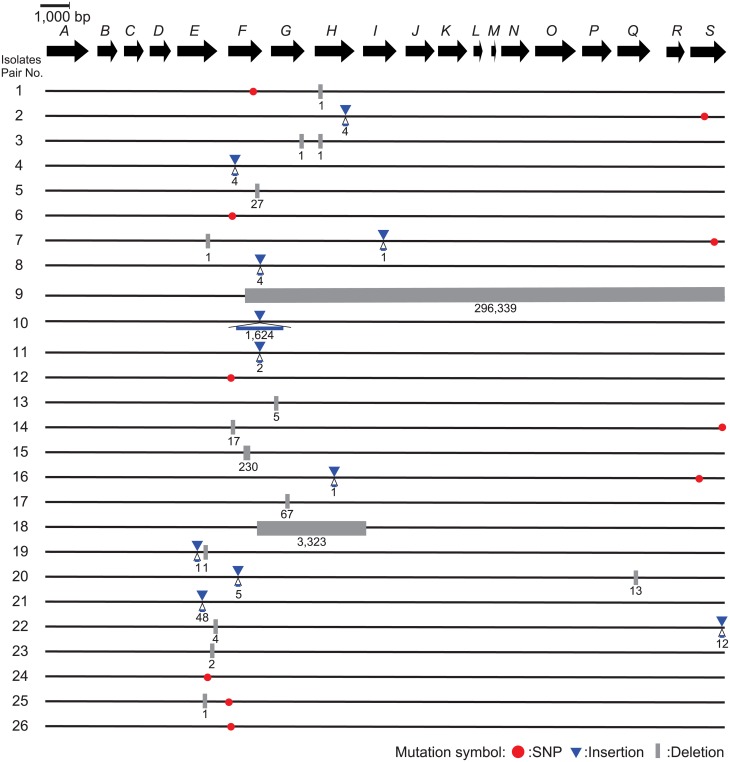
Nonsynonymous mutations and indels in the *cps* gene cluster between encapsulated and unencapsulated *S*. *suis* isolates in 26 pairs. The genetic organization of the *cps* gene cluster (from *cps2A* to *cps2S*) is shown as a sequence of the arrows located by transcriptional directions with the identifiers such as “A” for “*cps2A*”. The mutations between encapsulated and unencapsulated isolates in each pair are shown along with the genetic map, with the following symbols: red circles for nonsynonymous mutations, blue triangles for insertions, and gray rectangles for deletions along with the nucleotide length of the mutations.

The nucleotide sequences of the *cps* gene cluster in pairs no. 1, 2, 3, 9, 10, 11, and 15 were determined by Sanger sequencing. The length of indels resulting in frameshift mutations ranged from 1 to 4 nucleotides in 4 pairs (pairs no. 1, 2, 3, and 11), whereas indels with sizes spanning from 10^2^- to 10^4^-bp were found in the remaining 3 pairs (pairs no. 9, 10, and 15) (Fig 3). We next complemented *in trans* the unencapsulated isolates of pairs no. 1, 2, 3 and 11 with expression vectors of *cps* genes. Construct pCps2H was used for those of the pairs no. 1, 2, and 3, which had indels in the *cps2H* gene, while construct pCps2E was used to complement pair no. 11, which had mutations in the *cps2E* gene. All transformants showed a positive reaction in the co-agglutination test using anti-serotype 2 serum, indicating restoration of capsule expression.

### Occurrence of mutations in single-copy common genes, in the *cps* gene cluster, and in MGEs

Mapping results of 52 isolates using CLC Genomics Workbench are listed in S3 Table. The nucleotide sequences of single-copy common genes and the *cps* gene cluster were compared within each pair. The analysis was verified by the consistency of the mutation profiles in the Sanger and MiSeq-based methods. No mutation was found in the *ccpA* gene that regulated the production of CPs, and nonsynonymous mutations found between the pairs were depicted in Fig 3 [54].

**Fig 4:**
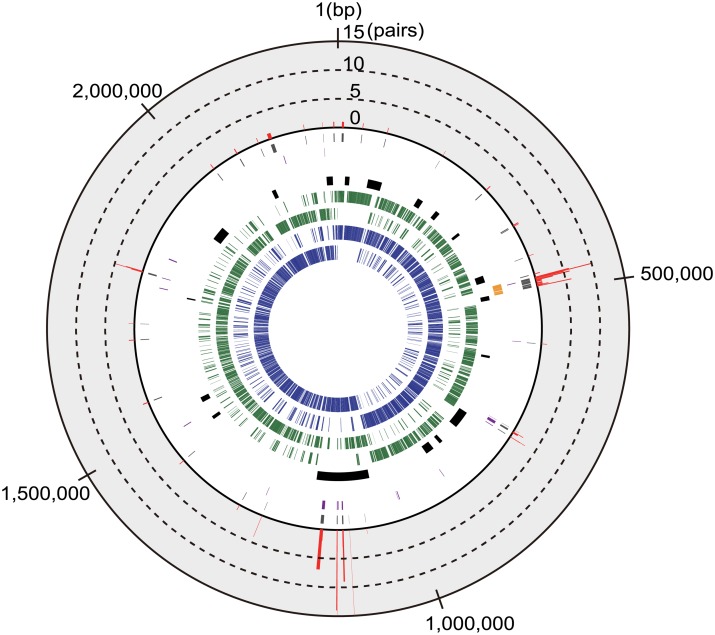
The genome structure of the ST28 *S*. *suis* strain NSUI002. The following information on the NSUI002 genome is shown from the innermost circle: protein-coding genes transcribed clockwise (1^st^ track, blue) and counterclockwise (2^nd^, blue), common genes that were shared among NSUI002 and all ST28 *S*. *suis* isolates, transcribed clockwise (3^rd^, green) and counterclockwise (4^th^, green), MGEs (5^th^, black), and genes in the *cps* gene cluster (6^th^, yellow). The common genes in which synonymous and nonsynonymous mutations were detected are shown in the 7^th^ (purple) and 8^th^ (gray) tracks, respectively. The outermost bar graph for the common genes in the 9^th^ track (red) shows the number of pairs in which nonsynonymous mutations were detected. No common gene exhibited a number of pairs more than 15, the maximum value of the graph.

There were differences between ST1 and ST28 pairs in the number of genome-wide mutations identified. Only a few nonsynonymous mutations were identified in the ST1 pairs, whereas the ST28 pairs had a large number of unique nonsynonymous mutations (Fig 4, S4 and S5 Tables). Indeed, the mean mutation frequencies in all single-copy common genes between ST1 and ST28 were calculated to be 2.70 × 10^−7^ and 3.25 × 10^−5^, respectively. At the nucleotide level, most of the mutations were unique for each pair. All the pairs showed SNPs within the *cps* gene cluster. In addition, SNPs within a particular MGE (position 1,054,531–1,178,032 bp of the reference genome NSUI002) were found in many pairs. This MGE contains genes related to conjugative transposons (Fig 4, S6 Table).

**Fig 5:**
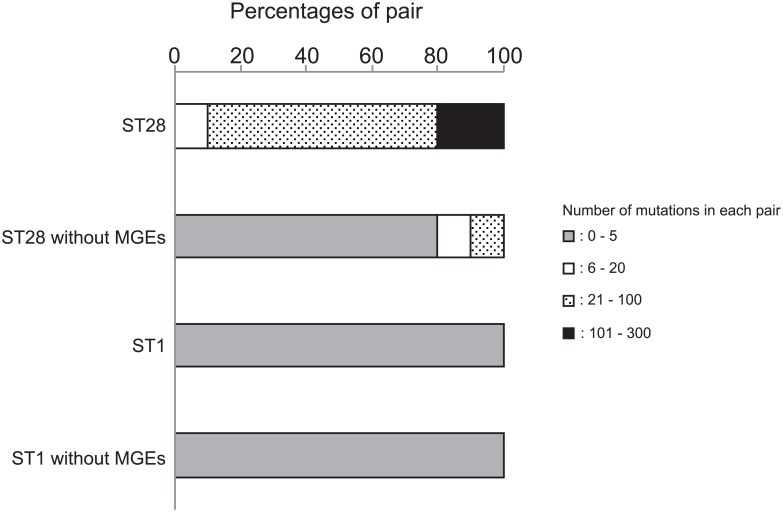
The proportion of each pair classified by number of mutations. The number of mutations was calculated in each of 26 pairs, and its distribution is shown in ST1 and ST28 isolates as a percentage of pairs, as follows: gray bars for pairs with 0–5 mutations, blank bars for 6–20, dotted bars for 21–100, and filled bars for 101–300. Percentages are shown in ST1 and ST28 clones when genes on MGEs are excluded from the calculation.

The maximum numbers of nonsynonymous mutations per gene were 3 and 15 in the ST1 and ST28 pairs, respectively. Among ST28 pairs, most genes with mutations were part of MGEs (Fig 4). Therefore, we excluded the single-copy common genes in MGEs and examined the profiles of mutations. The number of mutations in each pair ranged between 0 to 5, except for the genes within MGEs, which ranged between 21 to 302 (Fig 5). Thus, the mutation frequencies were amended to be 2.95 × 10^−7^ in ST1, and 3.25 × 10^−6^ in ST28.

## Discussion

In the present study, we demonstrated the coexistence of encapsulated and unencapsulated *S*. *suis* in the same endocarditis lesion. The capsule protects *S*. *suis* cells from the host immune response [24, 30, 55, 56]. *S*. *suis* is known, however, to escape the vaccine-immunity by means of capsule loss [57–59], such as reported previously for the capsule loss of *Haemophilus influenzae* [11, 12]. Unencapsulation can also afford *S*. *suis* cells some advantages, such as high adhesion and invasion of host cells [32–35]. This led us to hypothesize that encapsulated and unencapsulated *S*. *suis* “cooperate” by expressing both advantageous characteristics at lesions, and that each of these phenotypically distinct, but genetically very similar clonal subpopulations provides an advantage that results in persistence of the global population of the clone. Taken all together, our results seem to support the abovementioned hypothesis. However, we cannot disregard the alternative hypothesis that the unencapsulated cells in the endocarditis lesions may not have the capacity to participate in any significant way in inducing heart lesions, but they simply arise by their faster growth in the particular niche.

We obtained only unencapsulated isolates from 2 samples. Moreover, the ratio of both phenotypes varied among 26 samples. It is conceivable that the encapsulated and unencapsulated cells did not form a completely mixed jumble within the lesions but instead formed different layers of either encapsulated or unencapsulated cells. Assuming that the layer of unencapsulated cells was cut out and stamped on an agar medium, we can isolate only unencapsulated cells from the sample. Indeed, we always succeeded in isolating both phenotypes from the same lesion by cutting the samples in many pieces for stamp (our unpublished observation). We collected endocarditis samples from many geographically separated farms, and many of these unrelated samples harbored both encapsulated and unencapsulated phenotypes of the same clone of *S*. *suis* (Fig 1A). The high prevalence may suggest that occurrence of unencapsulated isolates does not require specific conditions and occur frequently in every farm.

We only detected ST1 and ST28 *S*. *suis* isolates in our sample (Fig 1B). Both ST1 and ST28 are prevalent in Japan [60]. ST1 is also prevalent worldwide and the isolates belonging to ST1 generally express a hemolysin known as suilysin [61]. ST28 has become prevalent in several countries, including the United States of America, Canada, and China [49, 62] and exhibits a broad range of virulence in porcine groups [49]. The very close genetic relationship of the isolates within each ST (Fig 2) suggests transmission of each lineage across distant farms by porcine transportations, similar to the transmission of *Salmonella* spp. from slaughterhouses to farms [63]. However, we were unable to find any relationships between the geographical location of the farms and STs of the isolates (Fig 1).

Some bacterial species have been known to cause polyphenotypic infections. Previous studies reported the coexistence of multiple *P*. *aeruginosa* clones that exhibit different antimicrobial resistance and colony formation. Because such phenotypic differences were observed after the establishment of infection and lesion formation, it was suggested that they differentiate *in situ* at lung lesions [6, 64, 65]. Since encapsulated and unencapsulated *S*. *suis* isolates in most pairs were phylogenetically closest to each other (Fig 2), the encapsulated and unencapsulated lineages may have differentiated in the farm, i.e., environment or host body. Bacteria can change their phenotype to adapt to the surrounding environment, such as genetic change in *P*. *aeruginosa*, or transcriptional regulation in *Streptococcus equi* [66]. In the present analyses of the *cps* gene cluster, all pairs of encapsulated and unencapsulated isolates had mutations that included single-nucleotide polymorphisms and indels (Fig 3). As reported in a previous study, in which capsule production by unencapsulated *S*. *suis* isolates was complemented with the intact *cps* genes of reference strains P1/7 and 89–1591 [21, 46], we also demonstrated here the complementation of capsule production in unencapsulated isolates with *cps* expression vectors. This result suggests that capsule expression by *S*. *suis* in endocarditis lesions was affected by genetic changes in the *cps* gene cluster.

The expression of capsule is generally affected by regulatory genes, such as *ccpA*. In the present study, we did not find any modification in the *ccpA* gene. Although there may be the other regulatory genes that affect expression of capsule, spontaneous mutations in the *cps* genes cluster are likely to be the main mechanism leading to unencapsulation. Indeed, all *cps* mutants examined to date had nucleotide changes in structural genes of enzymes for capsule production [21, 46]. Among the mechanisms that generate nucleotide changes, spontaneous mutations are a major cause that are frequently observed in various events such as errors in DNA replication [67]. In the mutation profiles of all single-copy common genes, the spontaneous mutations unique for a single pair were predominant throughout the genomes (Fig 4); however, there were mutations in the *cps* gene cluster that occurred in multiple pairs. This may have been due to the limitation of the examined isolates and a bias for the selection of the phenotypes. Mutation frequencies differed between the ST1 and ST28 pairs; however, the difference may be explained by the large number of mutations concentrated within a MGE in ST28 pairs (approximately 90% of mutations were within a MGE (Fig 4, S6 Table)). After the exclusion of the single-copy common genes within MGEs, the mutation frequency was almost the same to those in other *Streptococcus* spp. such as *S*. *equi* (5.22 × 10^−7^) [68], *S*. *pneuomoniae* (1.57 × 10^−6^) [69] and, *S*. *pyogenes* (1.1 × 10^−6^) [70]. Collectively, these findings suggest that mutations may spontaneously arise in the *cps* gene cluster; therefore, these mutations may alter the phenotypic change through capsule expression. On the other hand, the MGE found in ST28 contained two genomic regions that exhibited high nucleotide sequence similarity with *attL* and *attR*, parts of a conjugative element, as well as genes related to its transfer [71]. Although precise cause why the MGE carried more mutations than other genome regions remains unknown, we assume the region may be related to the cause of endocarditis, which will be revealed by genome-wide association study in the future [72].

In summary, we herein demonstrate that different phenotypes of genetically very closely related *S*. *suis* isolates were from the same endocarditis lesion. *S*. *suis* may adapt to its surrounding environment through the loss of capsule expression, and endocarditis lesion may be developed by dual phenotypes of a single clones. Although unencapsulated *S*. *suis* isolates are, in general, easily phagocytized by immune cells, it is possible that they persist and proliferate in the host by the assistance of encapsulated cells. In addition, a comparison of ST1 and ST28 suggested that the isolates of different STs employed various mechanisms by which they adapt to the surrounding environment. Future studies about coexistence of different phenotypes, which may enable them to persist in endocarditis lesions, and experimental reproduction of endocarditis with both phenotypes, will provide insights into the pathogenicity, ecology, diversity and evolution of *S*. *suis*.

## Supporting Information

S1 Table *E*. *coli* strains and plasmids used in this study. (DOCX)

S2 Table Primers used to construct *cps* gene expression vectors. (DOCX)

S3 Table Mapping and assembly results of isolates. (XLSX)

S4 Table Mutations in single-copy common genes of ST28 isolates. (XLSX)

S5 Table Mutations in single-copy common genes in ST1 isolates. (XLSX)

S6 Table Gene contents of the MGE where the SNPs were most abundant. (XLSX)
