# Supplementary material for: Comparative Genome Analyses of Streptococcus suis Isolates from Endocarditis Demonstrate Persistence of Dual Phenotypic Clones
Source: PLoS One. 2016 Jul 19;11(7):e0159558. doi: 10.1371/journal.pone.0159558 (PMC4951133; doi:10.1371/journal.pone.0159558)
Supplement: S1 Table — (DOCX) [file pone.0159558.s001.docx]

S1 Table. *E. coli* strains and plasmids used in this study.

| Strain or plasmid | Relevant characteristics | Source of reference |
| --- | --- | --- |
| ***E. coli* strains** |  |  |
| Stellar^TM^ Competent Cells | F^-^, *emdA1*, *supE44*, *thi-1*, *recA1*, *relA1*, *gyrA96*, *phoA*, *φ80d* *lacZ*Δ*M15*,  Δ(*lacZYA* – *argF*) *U169*, Δ(*mrr* – *hsdRMS* – *mcrBC*), Δ*mcrA*, λ^-^ | Clontech Laboratories, Inc., CA, U.S. |
| MC1061 | Host for pMX1 derivatives | Casadaban & Cohen (39) |
| **Plasmids** |  |  |
| pMX1 | Spc^r^, pSSU1 *ori*, a *malX* promoter of *S. suis*, a derivative of pSET2 | Okura *et al*. (40) |
| pCps2E | pMX1 carrying an intact *cps2E* gene | This study |
| pCps2H | pMX1 carrying an intact *cps2H* gene | This study |
