# Supplementary material for: Comparative Genome Analyses of Streptococcus suis Isolates from Endocarditis Demonstrate Persistence of Dual Phenotypic Clones
Source: PLoS One. 2016 Jul 19;11(7):e0159558. doi: 10.1371/journal.pone.0159558 (PMC4951133; doi:10.1371/journal.pone.0159558)
Supplement: S2 Table — (DOCX) [file pone.0159558.s002.docx]

S2 Table. Primers used to construct *cps* gene expression vectors.

| Primer | Sequence (5´-3´) |
| --- | --- |
| pCps2E-F | TACCGAGCTCGAATTAATAGTGGAGGAGCT |
| pCps2E-R | GACGGCCAGTGAATTTCTTACTTACTTCCC |
| pCps2H-F | TACCGAGCTCGAATTGGTGGTGGCAAAAAAGAGA |
| pCps2H-R | GACGGCCAGTGAATTAAAATTACAAATAAAAGTAAACAAC |
